# Dobutamine stress cardiovascular magnetic resonance at 3 Tesla

**DOI:** 10.1186/1532-429X-10-44

**Published:** 2008-10-09

**Authors:** S Kelle, A Hamdan, B Schnackenburg, U Köhler, C Klein, E Nagel, E Fleck

**Affiliations:** 1 Department of Internal Medicine/Cardiology, Deutsches Herzzentrum Berlin, Germany; 2 Department of Radiology, Johns Hopkins University, USA; 3 Philips Healthcare, Hamburg, Germany; 4 Division of Imaging Sciences, King's College London, UK

## Abstract

**Purpose:**

The assessment of inducible wall motion abnormalities during high-dose dobutamine-stress cardiovascular magnetic resonance (DCMR) is well established for the identification of myocardial ischemia at 1.5 Tesla. Its feasibility at higher field strengths has not been reported. The present study was performed to prospectively determine the feasibility and diagnostic accuracy of DCMR at 3 Tesla for depicting hemodynamically significant coronary artery stenosis (≥ 50% diameter stenosis) in patients with suspected or known coronary artery disease (CAD).

**Materials and methods:**

Thirty consecutive patients (6 women) (66 ± 9.3 years) were scheduled for DCMR between January and May 2007 for detection of coronary artery disease. Patients were examined with a Philips Achieva 3 Tesla system (Philips Healthcare, Best, The Netherlands), using a spoiled gradient echo cine sequence. Technical parameters were: spatial resolution 2 × 2 × 8 mm^3^, 30 heart phases, spoiled gradient echo TR/TE: 4.5/2.6 msec, flip angle 15°. Images were acquired at rest and stress in accordance with a standardized high-dose dobutamine-atropine protocol during short breath-holds in three short and three long-axis views. Dobutamine was administered using a standard protocol (10 μg increments every 3 minutes up to 40 μg dobutamine/kg body weight/minute plus atropine if required to reach target heart rate). The study protocol included administration of 0.1 mmol/kg/body weight Gd-DTPA before the cine images at rest were acquired to improve the image quality. The examination was terminated if new or worsening wall-motion abnormalities or chest pain occurred or when > 85% of age-predicted maximum heart rate was reached. Myocardial ischemia was defined as new onset of wall-motion abnormality in at least one segment. In addition, late gadolinium enhancement (LGE) was performed. Images were evaluated by two blinded readers. Diagnostic accuracy was determined with coronary angiography as the reference standard. Image quality and wall-motion at rest and maximum stress level were evaluated using a four-point scale.

**Results:**

In 27 patients DCMR was performed successfully, no patient had to be excluded due to insufficient image quality. Twenty-two patients were examined by coronary angiography, which depicted significant stenosis in 68.2% of the patients. Patient-based sensitivity and specificity were 80.0% and 85.7% respectively and accuracy was 81.8%. Interobserver variability for assessment of wall motion abnormalities was 88% (κ = 0.760; p < 0.0001). Negative and positive predictive values were 66.7% and 92.3%, respectively. No significant differences in average image quality at rest versus stress for short or long-axis cine images were found.

**Conclusion:**

High-dose DCMR at 3T is feasible and an accurate method to depict significant coronary artery stenosis in patients with suspected or known CAD.

## Introduction

Several studies performed at different sites have demonstrated high diagnostic accuracy for high-dose dobutamine stress cardiovascular magnetic resonance (DCMR) at 1.5 Tesla to identify the presence of coronary artery stenoses and define the functional relevance of these lesions [1,2]. In patients with reduced image quality in stress echocardiography the superiority of DCMR was demonstrated [3,4].

Early DCMR studies performed at 1.5 Tesla used Turbo Gradient Echo Sequences (TGrE) [3-5]. Later, balanced steady-state free precession (SSFP) became the gold standard for cine CMR at 1.5 Tesla [6] and was also routinely used for DCMR [7,8]. However, increased B0 and B1 inhomogeneity at 3 Tesla compared to 1.5 Tesla led to problems with off-resonance artifacts, limiting the use of the current standard SSFP cardiac cine-imaging. Compared to SSFP sequences, TGrE techniques have the advantage of causing very few artifacts. However, one of the disadvantages of TGrE cine-images is the relatively low contrast between blood-pool and myocardium. Especially in the long-axis planes and in patients with impaired LV function the image quality is limited by reduced signal intensity of the blood due to saturation of blood flowing predominantly in plane, which may hinder LV endocardial border delineation and therefore functional assessment [9].

To overcome these limitations, we recently demonstrated that the application of an extracellular contrast agent before the acquisition of TGrE cine-images, improves image quality and blood-to-myocardium contrast in long-axis views and leads to better endocardial border delineation as compared with native long-axis cine-imaging at 3 Tesla [10]. Based on these results, we hypothesize that high-dose DCMR in combination with contrast agent administration at 3 Tesla is feasible by using TGrE cine sequences to detect significant coronary stenoses in patients with clinically suspected or known CAD. Thus, the purpose of our study was to prospectively determine the feasibility and diagnostic accuracy of high-dose DCMR using a 3 Tesla scanner for depicting clinically significant coronary artery stenoses in patients with suspected or known coronary artery disease (CAD).

## Materials and methods

### Patient population

The study was conducted in accordance with the standards of the Charité institutional review board. Patients with suspected or known coronary artery disease and recurrent angina, including patients with a history of myocardial infarction and previous percutaneous coronary intervention (PCI), were prospectively enrolled after giving written informed consent. Thirty patients (24 men and 6 women; mean age, 66 ± 9.3 years) scheduled for clinically indicated coronary angiography with suspected or known coronary artery disease were studied between January and May 2007. Patients were excluded if they had typical contraindications for CMR (non-compatible biometallic implants or claustrophobia) or administration of dobutamine or known arrhythmias [11,12]. Patients who had previously undergone coronary artery bypass graft surgery were not included (table 1).

**Table 1:** Patient demographics of 30 patients.

**Patient characteristics**	
Sex, F/M	6/24
Age, y	66 ± 9.4
Range	48 – 54
BMI, kg/m^2^	27 ± 30

**Medical history information**, n (%)	

Hypertension	24 (80)
Diabetes mellitus	11 (36.7)
Hyperlipoproteinemia	15 (50)
History of smoking	9 (30)
Family history of CAD	10 (33.3)
Suspected CAD	9 (30)
Known CAD	21 (70)
Previous PCI	17 (56.7)
Previous myocardial infarction	6 (20)

**Therapy following MRI**	

PCI	14 (46.7)
CABG	3 (10)

BMI = body mass index; CAD = coronary artery disease; PCI = percutaneous coronary intervention; CABG = coronary artery bypass graft.

All patients were instructed to refrain from β-blocker treatment 24 hours before CMR. The medical history of the patients was recorded at the time of CMR.

### CMR examination

All patients were examined in supine position using a 3 T whole-body MR system (Achieva 3T; Philips Healthcare, Best, The Netherlands) equipped with a Quasar Dual gradient system (80 mT/m, 200 T/m/s slew rate). A six-element cardiac synergy coil was used for signal detection. Cardiac synchronization was performed by a four electrode vector electrocardiogram, and image acquisitions were triggered on the R-wave [13]. The images were acquired in expiratory breath-holds.

### Imaging protocol

**Figure 1:**
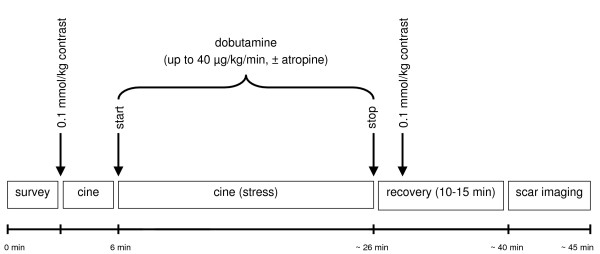
Time course of dobutamine stress CMR examination at 3 Tesla.

All patients received one 18 gauge intravenous line to allow administration of dobutamine and the contrast agent. The patients underwent a standardized CMR examination including the following steps, as demonstrated in figure 1:

1. Localization of the heart in the three standard planes (transversal, coronal and sagittal) using a rapid gradient echo sequence (multistack, multislice survey scan, TGrE, TR/TE/flip angle = 3.6 msec/1.7 msec/20°).

2. Cine-imaging of three short-axis views and long-axis views (four-chamber; two-chamber and three-chamber view). A spoiled gradient echo cine sequence was used. Technical parameters were: spatial resolution 2 × 2 × 8 mm^3^, 30 heart phases, spoiled gradient echo TR/TE = 4.5/2.6 msec, flip angle 15°. The breathhold duration per slice acquired was kept at approximately 10 sec. To accommodate the wide range of heart rates in the stress protocol (46 to 147 beats per minute), the number of k-space lines acquired for each cardiac phase per heart beat (TFE factor) had to be adjusted from 19 to 6. Accordingly, the acquired temporal resolution ranged from 83 to 39 msec, and was interpolated to 44 to 20 msec using a sliding window reconstruction. The three short-axis views were distributed to cover the heart at the basal, equatorial and apical position by adjusting the gap between the sections. Before the acquisition of cine short-axis images at rest was started, an intravenous bolus of 0.1 mmol/kg Gd-DTPA (Magnevist^®^, Schering, Berlin, Germany) at an injection rate of 2 ml/s followed by a flush of 20 ml of saline solution at the same rate was administered.

3. DCMR using the described cine sequence at every step of dobutamine administration. Dobutamine (10 to a maximum of 40 μg/min/kg body weight) was given for a total of 3 minutes at every step. Imaging was performed after 1 minute infusion of dobutamine at every step and required around 2 minutes. Up to 2 mg Atropine was administered if targeted heart frequency could not be reached. ECG rhythm and symptoms were monitored continuously and blood pressure was ascertained every 3 minutes. Standard termination criteria were used [7,14]. Adverse effects were recorded using a standardized reporting form. The stress test itself took around 20 minutes.

4. An additional bolus of 0.1 mmol/kg body weight Gd-DTPA given immediately after the last stress scan and followed by late gadolinium enhancement (LGE) 10–15 minutes later. LGE imaging was performed using an inversion prepared 3D spoiled gradient echo sequence (1.5 × 1.7 × 5 mm^3^). The whole protocol lasted around 45 minutes, similar in comparison to studies at 1.5 Tesla.

### Image analysis

Images were evaluated independently by two experienced readers blinded to the patients' history and angiographic results using a synchronized quad-screen image display and applying the standard scoring system (1 = normokinetic; 2 = hypokinetic; 3 = akinetic and 4 = dyskinetic). For determination of left ventricular ejection fraction and left ventricular end-systolic and end-diastolic volumes, three short-axis views and two long-axis views (four-chamber and two-chamber) were used. All myocardial segments were assigned to the three major coronary arteries in accordance with common definitions [15]. Ischemia was defined if ≥ 1 segment showed new wall motion abnormalities (increase of wall-motion score at stress) or if a biphasic response was demonstrated in areas with resting wall-motion abnormalities.

The image quality of each standard view (long and short axis) and, according to myocardial vascularization, of the four myocardial regions (anterior, lateral, inferior and septal) was rated on a 4-point scale for the visibility of the endocardial border (score 1 = poor or nondiagnostic; 2 = partial or moderate visibility; 3 = good visibility; and 4 = excellent visibility [10].

### Coronary angiography

Conventional coronary catheterization was performed within 7 ± 12 days after the CMR examination using a standard Judkins technique. A reduction of the luminal diameter ≥ 50% in one of the major epicardial coronary arteries or their major branches (≥ 2 mm) was defined as a hemodynamically significant stenosis. The stenosis severity was defined quantitatively by an experienced interventionalist blinded to the results of CMR.

### Statistical analysis

Statistical analysis was performed using SPSS for Windows (Statistical Package for Social Sciences (SPSS), release 12.0.1; Chicago, USA); for all continuous parameters, mean ± standard deviation is given. The paired Student's t-test was used to assess statistical significance of continuous variables. Sensitivity, specificity and diagnostic accuracy as well as negative and positive predictive values on a patient-based analysis were calculated according to standard definitions. For comparison of interobserver variability for the assessment of wall-motion abnormalities on a patient basis and in myocardial territories, kappa values were calculated. The Wilcoxon signed-rank test was used to evaluate the statistical correlation between the visual score of TgrE cine images at rest and at maximum stress. A p < 0.05 was considered statistically significant.

## Results

### Study group

For a detailed description of patients' hemodynamic data at rest and during stress see table 2.

**Table 2:** Hemodynamic data.

**Left ventricular function (rest)**	
LVEF, %	56.4 ± 6.8
LVEDV, ml	134.2 ± 31.7
LVESV, ml	60.9 ± 20.9

**Heart rate, bpm**	

At rest	63.3 ± 9.2
Maximum stress	123.1 ± 20.9

**Systolic blood pressure, mmHg**	

At rest	133.9 ± 20.7
Maximum stress	131.7 ± 29.2

**Diastolic blood pressure, mmHg**	

At rest	77.0 ± 11.9
Maximum stress	72.9 ± 15.6

**Heart rate-pressure product, bpm × mmHg**	

At rest	8505.2 ± 1956.9
Maximum stress	9709.6 ± 2541.7

Values are expressed as mean ± standard deviation. LVEF = left ventricular ejection fraction; LVEDV = left ventricular end-diastolic volume; LVESV = left ventricular end-systolic volume; bpm = beats per minute. Heart rate – pressure product is heart rate times systolic blood pressure.

In 3 (10%) of 30 patients the examinations had to be terminated at stress due to ECG-trigger problems which prohibited further data acquisition. One patient developed severe angina pectoris at maximum stress and requested termination of the examination; the symptoms resolved after administration of nitrates. Of the remaining 27 patients, 22 were available for comparative examination by coronary angiography. The average dosage of dobutamine and atropine was 36.3 ± 5.5 μg/min/kg body weight and 0.2 ± 0.4 mg, respectively.

### Diagnostic performance

**Figure 2:**
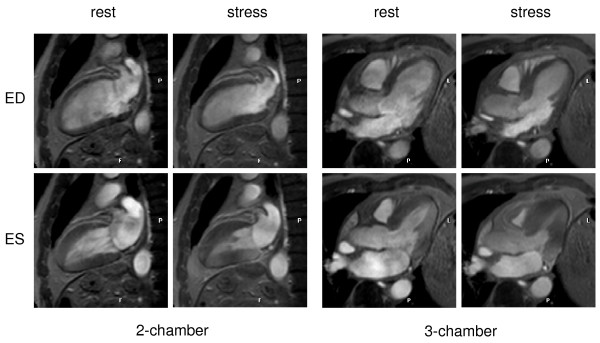
**Dobutamine stress CMR demonstrates no wall motion abnormalities at rest or at maximum stress.** In invasive coronary angiography, no CAD was found. ED = end-diastole; ES = end-systole.

**Figure 3:**
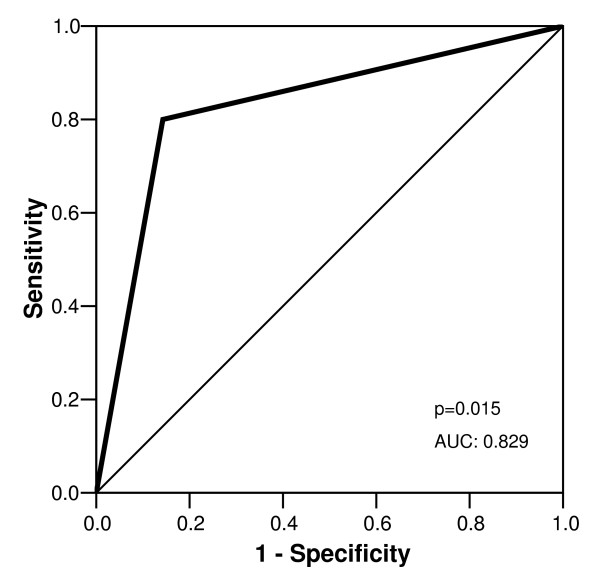
Sensitivity and specificity derived from ROC analysis for occurrence of dobutamine wall motion abnormalities in presence of coronary stenosis ≥50%. AUC indicates area under the curve.

**Figure 4:**
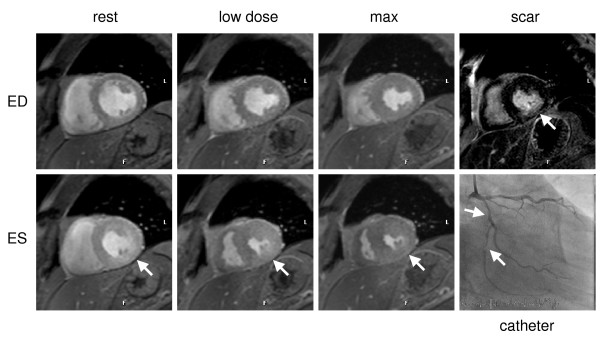
**Stress-induced ischemia (biphasic response) of the infero and infero-lateral wall in a patient with subtotal occlusion of the proximal and distal left circumflex artery (LCX) (white arrows).** Improvement at 20 ug/kg/min dobutamine of the wall motion abnormality at rest, however, decreased wall motion at maximum stress level. Late gadolinium enhancement revealed a 50% subendocardial infarction in this region.

Typical image quality at rest and stress are presented in figure 2. Hemodynamically significant coronary artery stenoses were present in 68.2% (15/22) of patients. The overall patient-based sensitivity, specificity and diagnostic accuracy for the detection of significant coronary artery stenosis (≥ 50%) were 80.0%; 85.7% and 81.8%, respectively (figure 3). Negative and positive predictive values were 66.7% and 92.3%, respectively. Figure 4 shows an example of a positive dobutamine stress study in a patient with obstructive coronary artery disease.

### Interobserver agreement

There was agreement in the determination of myocardial wall-motion abnormalities in 88% of the patients (κ = 0.760; p < 0.0001) on a per patient basis, in 100% of the LAD segments (κ = 1; p < 0.0001), 72% of LCX segments (κ = 0.426; p = 0.009) and 80% of the RCA segments (κ = 0.429; p = 0.022).

### Image quality at rest and maximum dobutamine stress

**Figure 5:**
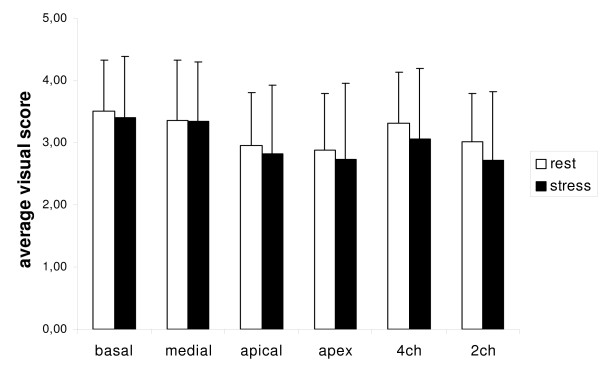
**Average visual score for endocardial border delineation for short-axis and long-axis cine-imaging at rest and maximum dobutamine stress.** Values are expressed as mean + one standard deviation. 4 ch = four-chamber view; 2 ch = two-chamber view. Between rest and stress cine-images no significant difference was demonstrated.

All examinations in both, rest and maximum stress cine-imaging yielded diagnostic image quality (minimum average score for a single slice orientation or myocardial region > 2.7). In short-axis views, the average image quality score differed at rest between the slice orientations, demonstrating the highest value for the basal short axis and decreasing to the lowest value for the apex. In every slice orientation there was a nonsignificant tendency towards decreased average image quality score at stress, compared to rest. In long-axis views; average image quality at stress tended to be reduced at peak stress in comparison to rest (Figure 5).

No significant difference could be found in average image quality score between rest and stress, arranged in accordance to the four myocardial regions (anterior, lateral, inferior and septal wall) (Table 3).

**Table 3:** Average image quality score in four myocardial regions.

	**Average visual score**		
**Myocardial region**	**Rest**	**Stress**	**p – value**

Anterior	3.19 ± 0.67	2.93 ± 0.96	0.198

Lateral	3.23 ± 0.66	3.10 ± 0.87	0.435

Inferior	3.12 ± 0.62	2.97 ± 0.92	0.395

Septal	3.42 ± 0.57	3.27 ± 0.89	0.413

## Discussion

In recent years, 3 Tesla CMR has become available and has demonstrated advantages over 1.5 Tesla over a broad range of applications, for example perfusion imaging [16,17]; late gadolinium enhancement [18]; peripheral MR angiography [19,20] and coronary magnetic resonance imaging [21].

The feasibility, accuracy, safety and prognostic value of high-dose DCMR at 1.5 Tesla in a wide range of patients is known to identify the presence of coronary artery stenoses and define the functional relevance of these lesions [1,2]. The assessment of inducible wall-motion abnormalities during high dose DCMR at 3 Tesla has not been systematically studied.

We have recently shown, that the administration of Gd-DTPA results in a significant improvement of contrast between blood and myocardium when using TGrE cine imaging at 3 Tesla [10]. In the current study we use a similar approach using Gd-DTPA. This resulted in a good image quality score in both rest and maximum stress cine-imaging examinations. The diagnostic image quality was yielded with a minimum average score for a single slice orientation or myocardial region > 2.7. Due to the use of TGrE cine-imaging and the relatively low flow velocity in the apical slice, the contrast between myocardium and blood was lower in the apical segments at rest and stress compared to the other myocardial segments. We found a slightly decreased average image quality score in every slice orientation at stress, compared to rest, but this was not statistically significant. By using this approach in all patients there was complete agreement in the determination of myocardial wall-motion abnormalities in 88% of the patients (κ = 0.760; p < 0.0001) on a per patient basis.

Recent reports have identified a possible link between a new scleroderma-like disorder, nephrogenic systemic fibrosis (NSF), and exposure to contrast agents containing gadolinium in patients with severe renal insufficiency. Therefore, the US Food and Drug Administration (FDA) and European medicines agencies recommend that in patients with severe renal impairment, agents containing gadolinium should be used only if clinically essential [22]. In our patient population, no subject presented severe renal impairment. However, to assess obstructive coronary artery disease, scar imaging should be performed. In recent studies in CAD and diabetes patients, the prognostic value of scar imaging was demonstrated [23,24]. In our study we did not report the results of scar imaging, because the readers were not blinded to the scar imaging results when they reviewed the wall-motion studies. Advantages of 3 T versus 1.5 T, especially for LGE, seem to be the possibility of a reduced contrast agent dose [25] and higher achievable spatial and temporal resolution [18].

ECG-trigger problems made it impossible to acquire data in three (10%) patients at peak stress. At higher field strength (3 Tesla), the magneto-hydrodynamic effect is enhanced and leads to an artifactual voltage overlayed on the T-wave of the ECG. This effect becomes more pronounced altering high-dose dobutamine stress, since flow in the aorta increases. This artifactual augmentation of the T-wave may mislead the R-wave detection algorithm, causing triggering on the T-wave instead of the R-wave. This problem may be overcome with sophisticated R-wave detection algorithms [13], which have been shown to be very reliable at rest [26]. One patient developed severe angina pectoris at maximum stress and requested termination of the examination; the symptoms resolved after administration of nitrates. This is within the range of the previously reported tolerance and safety profile of high-dose DCMR studies at 1.5 Tesla [27].

The diagnostic accuracy we report for high-dose DCMR at 3 Tesla in our study is within the range of previously published data on DCMR at 1.5 Tesla. A recently published meta-analysis pooled 13 CMR studies performed in 735 patients for stress-induced wall motion abnormalities and reported an overall sensitivity of 83% (95% confidence interval [25] 79 to 88%) and specificity of 0.86 (95% CI: 81 to 91%) for CAD at subject level. The prevalence of CAD in this patient group was 70.5% [2]. In our patient population with a similar prevalence of CAD of 73.5%, the sensitivity of 80% and specificity of 85.7% are comparable to the reported results.

However, given the more complex acquisition protocol requiring contrast agent administration and the tendentially lower diagnostic accuracy in comparison to own data at 1.5 Tesla, we currently see no advantage in using 3 Tesla over 1.5 Tesla for high-dose DCMR. Potentially, the combination with tagging techniques, which profit from the higher field strengths, may lead to improved results for 3 Tesla.

## Limitations

The major limitation of our study is the limited number of patients, which only allows a preliminary statement on diagnostic accuracy. In addition, the results represent a single center experience. Patients were only examined at 3 Tesla and no direct comparison to 1.5 Tesla is available. However, our aim was to establish a clinically robust approach for high-dose dobutamine CMR at 3 Tesla.

## Conclusion and future directions

High-dose dobutamine CMR at 3 Tesla is feasible and has been demonstrated to be an accurate method to detect hemodynamically significant coronary stenosis in this small patient group with suspected or known CAD. Larger trials are needed to establish the clinical role of CMR with 3 Tesla for the detection of CAD.

## Competing interests

The authors declare that they have no competing interests.

## Authors' contributions

SK, AH, BS, EF, UK and EN contributed to the study design, data interpretation, and manuscript review. SK and AH contributed to the data collection and data analysis. Finally, CK contributed to the manuscript preparation and review. All authors approved the final version of the manuscript submitted. All research support is acknowledged on the title page of the manuscript.
